# Impact of tight glycemic control and hypoglycemia after pediatric cardiac surgery on neurodevelopmental outcomes at three years of age: Findings from a randomized clinical trial

**DOI:** 10.1186/s12887-022-03556-z

**Published:** 2022-09-07

**Authors:** Anjali Sadhwani, Lisa A. Asaro, Caren S. Goldberg, Janice Ware, Jennifer Butcher, Michael Gaies, Cynthia Smith, Jamin L. Alexander, David Wypij, Michael S. D. Agus

**Affiliations:** 1 Departments of Psychiatry and Behavioral Sciences, Boston Children’s Hospital and Harvard Medical School, 300 Longwood Ave, Boston, MA 02115 USA; 2 Department of Cardiology, Boston Children’s Hospital, Boston, MA USA; 3 Division of Cardiology, C.S. Mott Children’s Hospital, Ann Arbor, MI USA; 4 Department of Pediatrics, University of Michigan Medical School, Ann Arbor, MI USA; 5 Department of Medicine, Boston Children’s Hospital and Harvard Medical School, Boston, USA; 6 Division of Pediatric Psychology, C.S. Mott Children’s Hospital and University of Michigan Medical School, Ann Arbor, MI USA; 7 Division of Medical Critical Care, Boston Children’s Hospital and Harvard Medical School, Boston, MA USA; 8 Department of Biostatistics, Harvard T.H. Chan School of Public Health, Boston, MA USA

**Keywords:** Cardiac surgery, Hypoglycemia, Glycemic control, Blood glucose, Neurodevelopment

## Abstract

**Background:**

Studies examining the impact of randomization As per standard instruction, city is required for affiliations; however, this information is missing in affiliation 6. Please check if the provided city is correct and amend if necessary. to tight glycemic control (TGC) and resultant hypoglycemia on later neurodevelopmental outcomes have produced mixed results. Our study examined this association in children undergoing cardiac surgery.

**Methods:**

Participants who were enrolled in the Safe Pediatric Euglycemia after Cardiac Surgery (SPECS) trial returned for neurodevelopmental (ND) follow-up between 30 to 42.5 months of age. ND outcomes were assessed using the Bayley Scales of Infant and Toddler Development, Third Edition. ND scores were compared between the TGC and standard care treatment groups and between patients with moderate to severe and no to mild hypoglycemia. As a secondary analysis, to increase sample size and power, we combined the three-year-old assessments with previously collected assessments done at < 30 months of age to further examine differences between groups longitudinally.

**Results:**

Among the 269 participants who completed neurodevelopmental evaluation (in-person testing or questionnaires) at three years of age (follow-up rate, 31%), there were no statistically significant differences in ND outcomes according to treatment group or hypoglycemia status. In the combined analysis of all evaluations (from 9 to 42.5 months of age), we found no treatment group differences. However, in these longitudinal analyses, children who experienced moderate to severe hypoglycemia had lower scores on the Bayley-III cognitive and motor domains compared to children with no to mild hypoglycemia.

**Conclusions:**

For infants undergoing cardiac surgery, there was no impact of tight glycemic control on neurodevelopmental outcomes. Moderate to severe hypoglycemia was associated with worse ND outcomes in longitudinal analyses.

**Trial registration:**

ClinicalTrials.gov NCT00443599. Registered: November 2016.

## Background

The immediate and short-term effects of using tight glycemic control (TGC) in critically ill pediatric patients have been investigated through multiple studies. Several meta-analyses have concluded that TGC was not associated with reduction of mortality or infections but instead was associated with an increased incidence of hypoglycemia [1–5].

Limited studies have examined the impact of randomization to TGC and resultant hypoglycemia on later neurodevelopmental outcomes, and results have been mixed. Biagas et al. (2020) found no differences in adaptive functioning at one-year after pediatric intensive care unit (ICU) discharge between patients randomized to lower vs. higher target glycemic control [6]. Results of a four-year follow-up for the Leuven randomized trial of TGC vs. standard care in critically ill patients ages 1–16 years admitted to the pediatric ICU noted improved motor coordination and cognitive flexibility in the TGC group [7]. Brief episodes of hypoglycemia were not associated with worse neurodevelopmental outcome; however, only intermittent blood glucose checking was employed [8]. On the other hand, results of our one-year-old neurodevelopmental (ND) follow-up from the Safe Pediatric Euglycemia after Cardiac Surgery (SPECS) trial, which used continuous glucose monitoring to track glycemia in children with congenital heart disease aged three years or younger undergoing cardiac surgery, found that children who experienced moderate to severe hypoglycemia (n = 8) had lower scores on the cognitive, language, and motor domains of development of the Bayley Scales of Infant and Toddler Development, Third Edition compared to patients with no to mild hypoglycemia, even after controlling for factors known to be associated with poorer ND outcomes [9].

Building upon our previous work, here we report on the ND follow-up of SPECS trial participants conducted at three years of age. The objective of this paper is to examine the association between randomization to TGC after pediatric cardiac surgery with ND outcomes at three years of age, a secondary outcome of the trial. In addition, we assess the relationship between hypoglycemia status and ND outcomes. Serial ND follow-up of this cohort is critical to assess whether the impact of hypoglycemia found at one year follow-up is also seen at three years of age. In addition, to increase sample size and power, we combine the ND follow-up data across all ages (9 to 42.5 months) to examine the impact of randomization to TGC and hypoglycemia status on neurodevelopmental outcomes after controlling for other patient and medical factors known to be associated with poorer ND outcomes.

## Methods

### Participants

In this two-center (Boston Children’s Hospital and the University of Michigan C. S. Mott Children’s Hospital) prospective randomized clinical trial [10, 11] (ClinicalTrials.gov, NCT00443599), 980 children aged 0–36 months undergoing cardiac surgery with cardiopulmonary bypass were randomly assigned either to the TGC group, where normoglycemia (i.e., glucose level between 80 to 110 mg/dL) was maintained using an insulin dosing algorithm, or to the standard of care (STD) group, which did not employ a blood glucose target. Patients were randomized according to a permuted-block design with stratification according to center. Continuous glucose monitoring was performed in all patients, which alerted for impending or actual hypoglycemia. Hypoglycemia episodes were determined based on glucometer and/or laboratory blood glucose values and were categorized as mild (50–59 mg/dL [2.8–3.3 mmol/L]), moderate (40–49 mg/dL [2.2–2.7 mmol/L]), or severe (< 40 mg/dL [< 2.2 mmol/L]). The study was reviewed and approved by the institutional review boards of the two centers. The primary outcome of the SPECS trial was the rate of health care-associated infections in the cardiac ICU. Neurodevelopment follow-up at one and three years of age were added as secondary outcomes to the study protocol in December 2008 after 159 patients had been enrolled in the study. The study was not powered on these secondary outcomes. Enrollment in the SPECS trial began in September 2006 and ended in May 2012; follow-up was conducted from March 2009 to August 2015. A detailed description of the study design, results of the primary outcome analysis, and results of the one-year-old ND follow-up have been published previously [9–12].

### Neurodevelopmental Follow-up at Three Years of Age

Participants were invited for three-year-old ND follow-up if they were between 30 to 42.5 months of age at the time of follow-up and lived in the United States. Two experienced pediatric psychologists (A.S. and J.B.) blinded to treatment group assignment and hypoglycemia status conducted the in-person assessments. The Bayley Scales of Infant and Toddler Development, Third Edition (Bayley-III) [13], a gold standard measure used to assess toddler development up to 42 months 15 days of age, was administered. Parents also completed the Adaptive Behavior Assessment System, Second Edition (ABAS-II) [14], a standardized caregiver-reported questionnaire to assess adaptive functioning, and the Behavior Assessment System for Children, Second Edition (BASC-2) [15], a standardized caregiver-reported questionnaire to assess behavior and socio-emotional functioning of children. Standard composite scores (mean ± standard deviation, 100 ± 15) and subscale scores (10 ± 3) are reported for the Bayley-III and ABAS-II while T-scores of the four composite domains (50 ± 10) are reported for the BASC-2. In addition, parents completed the Ages and Stages Questionnaire, Third Edition (ASQ-3) [16], a developmental screener that uses pre-established score thresholds to evaluate risk of developmental delay in several domains of functioning. The percentage of patients ‘at-risk’ based on ASQ-3 thresholds are reported. Parents were mailed the ASQ-3 if the family agreed to participate in the study but was unable to come to the clinic for an in-person visit.

### Statistical analyses

Descriptive statistics were calculated, including mean values and standard deviations for continuous variables and frequency counts and percentages for categorical variables. Comparisons by treatment group and hypoglycemia status were made using linear regression or stratified Wilcoxon rank-sum tests for continuous variables or stratified exact tests for categorical variables, with adjustment for site a priori. Moderate and severe hypoglycemia were combined in these analyses, given the low number of patients who had severe hypoglycemia. Analyses exploring the association of moderate to severe hypoglycemia with ND outcomes excluded patients with genetic anomalies that have established patterns of associated developmental disabilities [17, 18].

As a secondary analysis, to increase sample size and power, we combined all ND assessments across all ages (9–42.5 months), including multiple assessments per patient, using longitudinal multivariable linear regression analysis with adjustment for site. We used regression analysis to evaluate the association of moderate to severe hypoglycemia with ND outcomes after controlling stepwise for other factors known to be associated with poorer ND outcomes [19]. These factors included age at surgery of 60 days or younger [12], Risk Adjustment in Congenital Heart Surgery (RACHS-1) category of ≥ 3 (or not assignable) [19], single ventricle physiology, premature birth, maternal education (high school diploma or lower vs associate’s degree or higher), prolonged cardiopulmonary bypass (≥ 150 min), deep hypothermic circulatory arrest, delayed sternal closure, treatment group (TGC or STD), and cardiac ICU duration of stay tertile (< 2, 2–4.99, or ≥ 5 days), as well as age at ND testing. As an exploratory analysis, we also examined the interaction of moderate to severe hypoglycemia with age at testing in our longitudinal regression analyses. We used generalized estimating equations in final regression analyses to account for correlation between repeated measures from individuals. Analyses were performed using SAS (version 9.4, SAS Institute, Cary, North Carolina).

## Results

**fig. 1:**
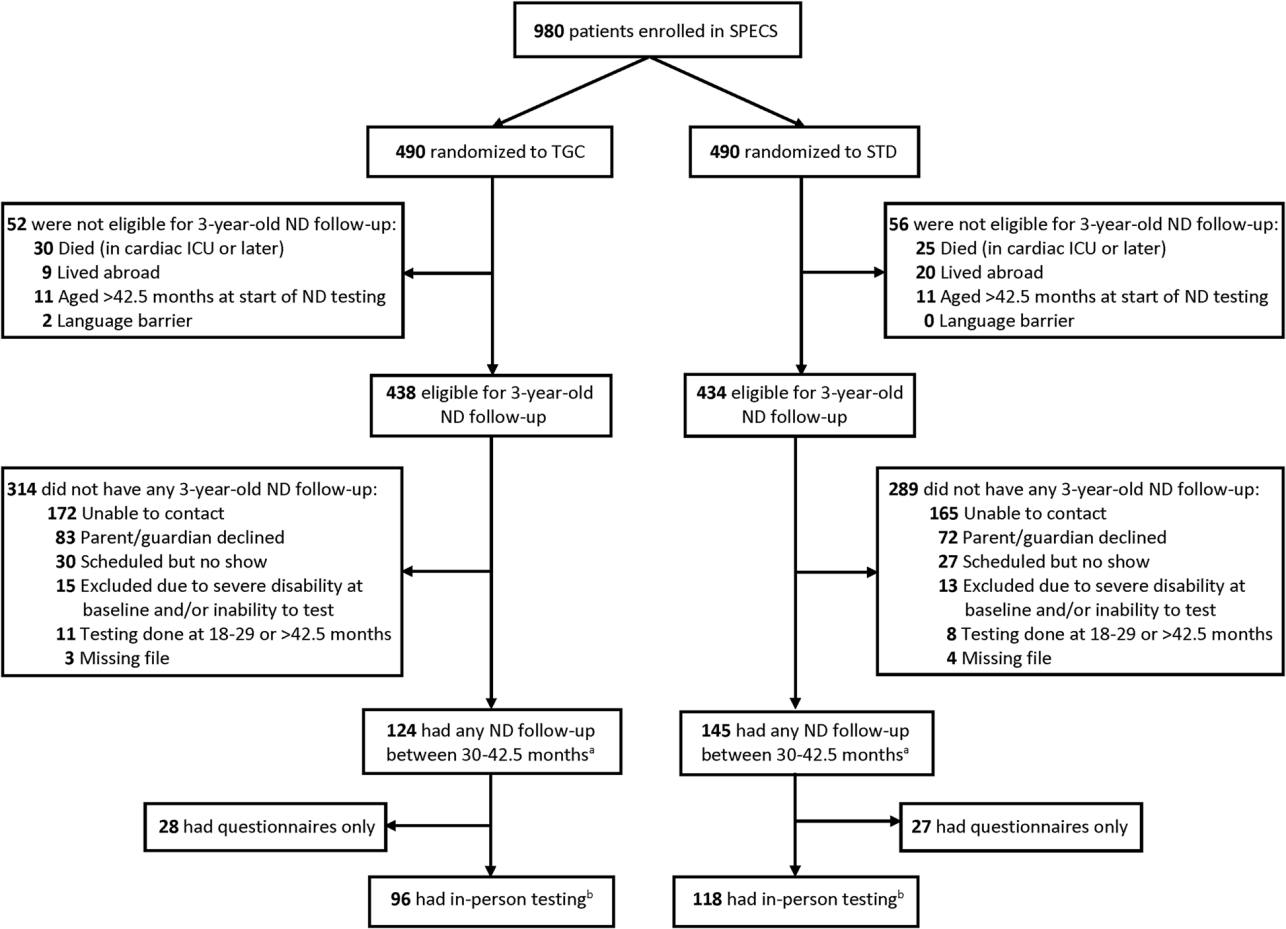
Flow chart of patients enrolled in the SPECS trial. *ND* Neurodevelopmental, *SPECS* Safe pediatric euglycemia after cardiac surgery, *STD* Standard care, *TGC* Tight glycemic control. 872 patients were eligible for 3-year-old follow-up, 269 had any follow-up between 30–42.5 months of age, and 214 had in-person testing. ^a^ 221 patients (97 TGC, 124 STD) without genetic anomaly. ^b^ 169 patients (72 TGC, 97 STD) without genetic anomaly

Of the 872 participants eligible for ND follow-up at three years of age, 269 participants (124 TGC, 145 STD) completed any ND follow-up (either in-person testing or parent-completed questionnaires) between 30 to 42.5 months of age (Fig. 1), at a median of 32 months after cardiac surgery (interquartile range, 28–36). There was a significant difference in participation rate by site (Boston 22% vs Michigan 47%; *p* < 0.001). Patients who returned for ND follow-up were more likely to be ≤ 60 days of age at the time of surgery (*p* = 0.008) and have single ventricle physiology (*p* = 0.01) than those who did not return (Table 1). For all other prerandomization, intraoperative, and postoperative characteristics, the two groups were comparable.

**Table 1:** Patient Characteristics According to Three-Year-Old Neurodevelopmental Follow-up Status

Characteristic	ND follow-up (*n* = 269)	No ND follow-up (*n* = 603)	*p*^a^
***Prerandomization characteristics***			
Enrolled in Boston, *n* (%)	128 (48)	442 (73)	< 0.001
Age at surgery, median (IQR), mo	3.9 (1.1–7.6)	4.9 (2.4–10.6)	0.02
≤ 60 d, *n* (%)	85 (32)	126 (21)	0.008
Female sex, *n* (%)	119 (44)	279 (46)	0.82
RACHS-1 category ≥ 3 or not assignable, *n* (%)	145 (54)	295 (49)	0.40
Single ventricle physiology, *n* (%)	66 (25)	85 (14)	0.01
Premature birth (< 37 weeks), *n* (%)^b^	35 (13)	89 (15)	0.45
Genetic anomaly, *n* (%)	48 (18)	117 (19)	0.77
***Intraoperative characteristics***			
Duration of cardiopulmonary bypass ≥ 150 min, *n* (%)	49 (18)	124 (21)	0.99
Deep hypothermic circulatory arrest, *n* (%)	45 (17)	77 (13)	0.19
Delayed sternal closure, *n* (%)	32 (12)	57 (9)	0.62
***Postoperative characteristics***			
Tight glycemic control group, *n* (%)	145 (54)	289 (48)	0.11
Treated with insulin therapy in the cardiac ICU, *n* (%)	124 (46)	283 (47)	0.60
Time-weighted blood glucose average, mean ± SD, mg/dL	120 ± 21	120 ± 22	0.88
Moderate to severe hypoglycemia (< 50 mg/dL), *n* (%)	13 (5)	34 (6)	0.41
Cardiac ICU duration of stay, *n* (%), d			0.10
< 2	95 (35)	221 (37)	
2–4.99	104 (39)	211 (35)	
≥ 5	70 (26)	171 (28)	

*ND* Neurodevelopmental, *RACHS-1* Risk adjustment in congenital heart surgery

^a^*p* values for the comparison between groups were calculated with the use of stratified exact tests for categorical variables, stratified Wilcoxon rank-sum test for age at surgery, or linear regression for time-weighted blood glucose average, with adjustment for site

^b^ In the ND follow-up group, premature birth not available for 1 patient (adopted, birth history unknown)

Among the 269 participants with three-year-old ND follow-up, there were no statistically significant differences between treatment groups in prerandomization or intraoperative characteristics (Table 2). Postoperatively, participants in the TGC group were more likely to be treated with insulin therapy (*p* < 0.001) and had lower time-weighted blood glucose averages (*p* < 0.001) than participants in the STD group. Moderate to severe hypoglycemia was experienced by 8 patients in the TGC group and 5 patients in the STD group (*p* = 0.39).

**Table 2:** Characteristics of Patients with Three-Year-Old Neurodevelopmental Follow-up According to Treatment Group

Characteristic	Tight glycemic control (*n* = 124)	Standard care (*n* = 145)	*p*^a^
***Prerandomization characteristics***			
Enrolled in Boston, *n* (%)	55 (44)	73 (50)	0.33
Age at surgery, median (IQR), mo	3.8 (1.2–7.1)	4.2 (1.0–7.7)	0.87
≤ 60 d, *n* (%)	38 (31)	47 (32)	0.79
Female sex, *n* (%)	60 (48)	59 (41)	0.22
RACHS-1 category ≥ 3 or not assignable, *n* (%)	69 (56)	76 (52)	0.71
Single ventricle physiology, *n* (%)	30 (24)	36 (25)	0.77
Premature birth (< 37 weeks), *n* (%)^b^	14 (11)	21 (15)	0.47
Genetic anomaly, *n* (%)^c^	27 (22)	21 (14)	0.15
Maternal education: high school diploma or lower, *n* (%)^d^	32 (27)	32 (23)	0.39
***Intraoperative characteristics***			
Duration of cardiopulmonary bypass ≥ 150 min, *n* (%)	16 (13)	33 (23)	0.055
Deep hypothermic circulatory arrest, *n* (%)	16 (13)	29 (20)	0.14
Delayed sternal closure, *n* (%)	10 (8)	22 (15)	0.09
***Postoperative characteristics***			
Treated with insulin therapy in the cardiac ICU, *n* (%)	118 (95)	6 (4)	< 0.001
Time-weighted blood glucose average, mean ± SD, mg/dL	114 ± 13	125 ± 25	< 0.001
Moderate to severe hypoglycemia (< 50 mg/dL), *n* (%)	8 (6)	5 (3)	0.39
Cardiac ICU duration of stay, *n* (%), d			0.81
< 2	41 (33)	54 (37)	
2–4.99	54 (44)	50 (34)	
≥ 5	29 (23)	41 (28)	

*RACHS-1* Risk adjustment in congenital heart surgery

^a^*p* values for the comparison between treatment groups were calculated with the use of stratified exact tests for categorical variables, stratified Wilcoxon rank-sum test for age at surgery, or linear regression for time-weighted blood glucose average, with adjustment for site

^b^ One patient was very preterm (gestational age 29 weeks), while the remaining 34 patients were moderate to late preterm (32 to 36 weeks). Premature birth not available for 1 standard care patient (adopted, birth history unknown)

^c^ Genetic anomalies include trisomy 21 (*n* = 31), 22q11 (n = 8), Charge association (*n* = 2), 10q24.32 (*n* = 1), Alagille syndrome (*n* = 1), trisomy X (*n* = 1), Williams syndrome (*n* = 1), and other specific genetic anomalies (8p23.1 deletion, abnormal MLL2, Xq21.31 deletion; n = 1 each)

^d^ Maternal education level not available for 6 tight glycemic control and 4 standard care patients

In-person ND testing was conducted on 96 participants in the TGC group and 118 participants in the STD group while for 55 patients, parents completed only questionnaires (28 TGC, 27 STD, *p* = 0.54). There were no statistically significant differences between the TGC and STD groups on the different domains of developmental functioning using the Bayley-III, adaptive functioning using the ABAS-II, or measures of behavioral and socio-emotional functioning using the BASC-2 (Table 3). The treatment groups were also comparable in terms of percentage at-risk for concerns across the different domains on the ASQ-3 (Table 3) and in the percentage of patients who received early intervention services (TGC 65% vs STD 60%; *p* = 0.37).

**Table 3:** Three-Year-Old Neurodevelopmental Outcomes According to Treatment Group

Outcome	Tight glycemic control (*n* = 124)	Standard care (*n* = 145)	*p*^a^
***Bayley-III scores***^***b***^	**(*n* = 96)**	**(*n* = 118)**	
Age at testing, mean ± SD, mo	37.0 ± 2.7	37.1 ± 2.6	0.93
Composite scores, mean ± SD			
Cognitive	94.6 ± 14.7	95.7 ± 13.2	0.66
Language	97.8 ± 19.9	99.8 ± 17.2	0.44
Motor	89.3 ± 17.4	92.6 ± 16.9	0.19
Subscale scores, mean ± SD			
Cognitive	8.9 ± 2.9	9.1 ± 2.6	0.66
Receptive communication	9.9 ± 3.4	10.2 ± 2.8	0.52
Expressive communication	9.2 ± 3.6	9.6 ± 3.3	0.36
Fine motor	8.9 ± 3.0	9.4 ± 3.0	0.27
Gross motor	7.5 ± 3.1	8.1 ± 3.0	0.16
***ABAS-II composite scores,***mean ± SD^c^	**(*n* = 84)**	**(*n* = 100)**	
General Adaptive Composite	89.2 ± 21.6	91.7 ± 19.4	0.42
Conceptual	95.0 ± 21.2	96.5 ± 19.5	0.64
Social	91.6 ± 20.4	95.5 ± 17.3	0.17
Practical	85.1 ± 19.8	87.3 ± 17.0	0.42
***BASC-2 composite T-scores,***mean ± SD^d^	**(*n* = 93)**	**(*n* = 116)**	
Externalizing	45.7 ± 9.7	46.8 ± 9.3	0.40
Internalizing	47.5 ± 10.2	47.6 ± 9.3	0.99
Behavioral symptoms	46.6 ± 9.4	46.7 ± 8.6	0.98
Adaptive skills	48.9 ± 9.7	50.4 ± 9.5	0.27
***ASQ-3, at risk,****n* (%)^e^	**(*n* = 112)**	**(*n* = 131)**	
Communication	25 (22)	24 (18)	0.52
Gross motor	30 (27)	30 (23)	0.55
Fine motor	23 (21)	16 (12)	0.11
Problem solving	28 (25)	24 (18)	0.27
Personal-social	30 (27)	23 (18)	0.09

*ABAS-II* Adaptive behavior assessment system, second edition, *ASQ-3* Ages and stages questionnaire, third edition, *BASC-2* Behavior assessment system for children, second edition, *Bayley-III* Bayley scales of infant and toddler development, third edition

^a^*p* values for the comparison between treatment groups were calculated with the use of linear regression or stratified exact tests with adjustment for site, as appropriate

^b^ Bayley-III cognitive and language composite scores and cognitive and receptive language subscale scores were not available for 1 tight glycemic control patient. Bayley-III motor composite score and gross motor subscale score were not available for 1 tight glycemic control patient

^c^ ABAS-II general adaptive composite and social scores were not available for 1 tight glycemic control patient. ABAS-II general adaptive composite and practical scores were not available for 1 standard care patient

^d^ BASC-2 internalizing composite T-score was not available for 2 tight glycemic control patients. BASC-2 internalizing and adaptive skills composite T-scores were not available for 1 standard care patient

^e^ ASQ gross motor score was not available for 1 tight glycemic control patient

After excluding 45 children with a genetic anomaly who returned for in-person testing, we found that more patients who experienced moderate to severe hypoglycemia (4 TGC, 2 STD) had surgery within the first 60 days of life (*p* = 0.03), had complex heart disease (*p* = 0.03), and had a cardiac ICU duration ≥ 5 days (*p* = 0.003; Table 4) than patients who did not experience hypoglycemia. Four of those patients (2 TGC, 2 STD) had one hypoglycemia episode while two patients (2 TGC) had two episodes*.*

**Table 4:** Patient Characteristics and Three-Year-Old Neurodevelopmental Outcomes for Patients Without a Genetic Anomaly According to Hypoglycemia Status

Variable	Moderate to severe hypoglycemia (*n* = 6)	No to mild hypoglycemia (*n* = 163)	*p*^a^
***Patient characteristics***			
Age at surgery, median (IQR), mo	0.3 (0.2–0.6)	3.8 (0.6–7.4)	0.08
≤ 60 d, *n* (%)	5 (83)	55 (34)	0.03
RACHS-1 category ≥ 3 or not assignable, *n* (%)	6 (100)	84 (52)	0.03
Single ventricle physiology, *n* (%)	1 (17)	43 (27)	0.67
Premature birth (< 37 weeks), *n* (%)^b^	2 (33)	17 (10)	0.14
Maternal education: high school diploma or lower, *n* (%)^c^	2 (33)	37 (23)	0.63
Duration of cardiopulmonary bypass ≥ 150 min, *n* (%)	3 (50)	31 (19)	0.07
Deep hypothermic circulatory arrest, *n* (%)	1 (17)	29 (18)	0.99
Delayed sternal closure, *n* (%)	2 (33)	18 (11)	0.15
Tight glycemic control, *n* (%)	4 (67)	68 (42)	0.40
Cardiac ICU duration of stay, *n* (%), d			0.003
< 2	0	65 (40)	
2–4.99	1 (17)	60 (37)	
≥ 5	5 (83)	38 (23)	
***Bayley-III scores***^***d***^			
Age at testing, mean ± SD, mo	38.2 ± 2.6	36.9 ± 2.6	0.15
Composite scores, mean ± SD			
Cognitive	93.0 ± 9.1	99.2 ± 11.8	0.27
Language	101.4 ± 13.5	105.0 ± 14.5	0.57
Motor	87.7 ± 20.4	96.6 ± 13.8	0.15
Subscale scores, mean ± SD			
Cognitive	8.6 ± 1.8	9.8 ± 2.4	0.27
Receptive communication	10.8 ± 2.2	11.0 ± 2.5	0.86
Expressive communication	9.2 ± 2.5	10.6 ± 2.7	0.18
Fine motor	9.0 ± 3.0	10.0 ± 2.5	0.41
Gross motor	6.8 ± 4.2	8.8 ± 2.5	0.07

*Bayley-III* Bayley scales of infant and toddler development, third edition, *RACHS-1* Risk adjustment in congenital heart surgery

^a^*p* values for the comparison between groups were calculated with the use of linear regression for continuous variables (except stratified Wilcoxon rank-sum test for age at surgery) or stratified exact tests for categorical variables, with adjustment for site

^b^ Premature birth not available for 1 patient with no to mild hypoglycemia (adopted, birth history unknown)

^c^ Maternal education not available for 4 patients with no to mild hypoglycemia

^d^ Bayley-III cognitive and language composite scores and cognitive and receptive language subscale scores were not available for 1 patient with moderate to severe hypoglycemia. Bayley-III motor composite score and gross motor subscale score were not available for 1 patient with no to mild hypoglycemia

In terms of ND testing at three years of age, participants with moderate to severe hypoglycemia had lower scores on the gross motor domain of the Bayley-III than children with no to mild hypoglycemia; however, these results did not reach statistical significance (6.8 ± 4.2 vs 8.8 ± 2.5; *p* = 0.07).

We also performed a longitudinal analysis which included all assessments for study patients without a genetic anomaly who returned for any ND follow-up from 9 to 42.5 months of age to examine treatment group differences and to assess the impact of hypoglycemia on ND outcomes over time. A total of 250 patients without a genetic anomaly returned at any time point, of whom 148 had one assessment, 101 had two assessments, and one patient had three assessments. Of these 250 patients, 10 patients had moderate to severe hypoglycemia (8 TGC, 2 STD), including 2 patients with single ventricle physiology and 4 patients who had two assessments each. Adjusting for site, there were no differences in Bayley-III composite or subscale scores by treatment group when analyzing the data combined across all ages (data not shown); however, children with moderate to severe hypoglycemia scored 6.4 points lower on the Bayley-III cognitive composite (*p* = 0.04), 8.5 points lower on the language composite (*p* = 0.08), and 11.0 points lower on the motor composite (*p* = 0.04). After controlling for other risk factors associated with worse outcomes, children who experienced moderate to severe hypoglycemia continued to have lower scores on the Bayley-III cognitive (6.7 points; *p* = 0.04) and motor composite (11.2 points; *p* = 0.04) compared with children with no to mild hypoglycemia (Table 5). In addition to hypoglycemia status, older age at testing was associated with lower cognitive scores but higher language and motor scores. Lower maternal education and cardiac ICU duration ≥ 5 days were associated with lower language scores, while single ventricle physiology was associated with lower motor scores. We explored for interactions between moderate to severe hypoglycemia and age at testing; the interaction term was significant (*p* = 0.01) when added to the multivariable model for language score only (data not shown). The effect of moderate to severe hypoglycemia on language score was greater at 12 months of age (–9.5 points, 95% CI –20.6 to 1.6) than at 36 months of age (0.2 points, 95% CI –9.4 to 9.9).

**Table 5:** Longitudinal Multivariable Regression to Evaluate the Impact of Hypoglycemia Status and Other Factors on Neurodevelopmental Outcomes (9 to 42.5 Months) in Patients Without a Genetic Anomaly (*n* = 353 assessments of 250 patients)

Bayley-III Composite Score	Covariates	Beta Coefficient (95% CI)	*p*^a^
Cognitive^b^	Moderate to severe hypoglycemia	–6.7 (–13.1 to –0.4)	0.04
	Age at testing (per month)	–0.2 (–0.3 to –0.1)	< 0.001
Language^c^	Moderate to severe hypoglycemia	–5.1 (–14.9 to 4.7)	0.31
	Maternal education: high school diploma or lower	–5.3 (–8.9 to –1.6)	0.005
	Cardiac ICU duration of stay, d		
	< 2	Reference	
	2–4.99	–3.0 (–6.7 to 0.8)	0.12
	≥ 5	–7.2 (–10.8 to –3.6)	< 0.001
	Age at testing (per month)	0.3 (0.2 to 0.4)	< 0.001
Motor^d^	Moderate to severe hypoglycemia	–11.2 (–21.7 to –0.8)	0.04
	Single ventricle physiology	–4.2 (–7.6 to –0.7)	0.02
	Age at testing (per month)	0.2 (0.1 to 0.3)	< 0.001

*Bayley-III* Bayley scales of infant and toddler development, third edition, *CI* Confidence interval

^a^*p* values were calculated with the use of multivariable linear regression and generalized estimating equations to account for correlation between repeated measures from individuals. Coefficients for intercept and site are not reported

^b^*n* = 352 assessments of 249 patients, adjusted R^2^ = 0.06

^c^*n* = 345 assessments of 248 patients, adjusted R^2^ = 0.17

^d^*n* = 352 assessments of 249 patients, adjusted R^2^ = 0.06

## Discussion

In this two-center randomized clinical trial examining the impact of randomization to TGC and resultant hypoglycemia on the neurodevelopmental outcomes of infants and young children undergoing congenital heart surgery, we found that, consistent with our one-year-old ND follow-up findings [9], randomization to TGC was not associated with worse ND outcomes at three years of age. There were no differences between the TGC and STD groups on any standardized measures of developmental, adaptive, behavioral, or socio-emotional functioning. The results of our study are in contrast to the results obtained in Leuven’s randomized trial of TGC vs. standard care in which children in the TGC group had improved neurodevelopmental outcomes [7]. There were also no statistically significant differences by hypoglycemia status at three years of age, though our analyses were likely underpowered given the small sample size. In addition, in our secondary analysis, after combining ND follow-up from the SPECS study across all ages and controlling for other risk factors known to be associated with worse ND outcomes in children with congenital heart disease, we found that children who experienced moderate to severe hypoglycemia had lower scores on the cognitive and motor domains compared to children with no to mild hypoglycemia.

Our findings on the association of hypoglycemia with worse ND outcomes persisted in the cognitive and motor domains despite the number of patients with moderate to severe hypoglycemia in our study cohort being small. This is consistent with the growing body of research that has documented the impact of hypoglycemia on the developing brain [20–23]. Children with hypoglycemia are known to have white matter and cortical abnormalities which impact development [20]. The effects of hypoglycemia on neurodevelopment emerge in the early childhood period impacting the visual-motor domain and executive functioning [21] and persist through mid-childhood impacting literacy and learning [22, 23]. It is also possible that hypoglycemia continues to have long-term effects in the cognitive and motor domains and impacts higher-level skills that do not emerge until later phases of development, and hence serial follow-up into adolescence is recommended [24].

Our study has important implications for critical care management. Hypoglycemia is a potentially modifiable factor impacting neurodevelopment. The results of our study add to the literature by highlighting the ongoing need for close glucose monitoring during critical illness to avoid prolonged hypoglycemia. In addition, ongoing long-term ND monitoring to assess the functional impact of hypoglycemia is critical given the limited predictive value of ND testing for later development [24].

Our study is limited by the significant attrition rate and the small number of patients without a genetic anomaly experiencing moderate or severe hypoglycemia who were brought for ND follow-up at any age (10 patients). There also may be a potential selection bias in those who returned based on geographic location of the families and on the severity of medical (e.g., more likely to have single ventricle physiology) and developmental impairment in the children which may affect the generalizability of the study. In addition, the results of our study are confined to children who have undergone cardiac operation and may not be generalizable to other populations with complex medical needs. Hypoglycemia episodes were determined based on glucometer and/or laboratory blood glucose values. Since those values were intermittent, it was not possible to definitively measure duration of hypoglycemia. We also did not collect data on timing and duration of early intervention services to assess its potential effect on ND outcomes. In addition, the ASQ is a developmental screening tool completed by parents and is not as comprehensive as an in-person evaluation conducted by a clinician. Finally, while we adjusted for several factors known to be associated with ND outcomes, our exploratory multivariable linear regression models explained a low proportion of the variation in ND scores and may not have included other important events (e.g., cardiac arrest) that possibly modify the association between hypoglycemia and outcome. Our findings should be viewed as preliminary and be interpreted with caution given the small number of patients who experienced moderate to severe hypoglycemia. Future research should explore the potential association of hypoglycemia with long-term ND outcomes with a larger sample.

## Conclusions

Our study shows that, for infants undergoing cardiac surgery, randomization to tight glycemic control did not impact ND outcomes at three years of age. Resultant hypoglycemia status was also not associated with neurodevelopment, though our analyses were likely underpowered. However, when combining ND outcomes across the first three years of life, we found that moderate to severe hypoglycemia was associated with worse cognitive and motor outcomes, even after controlling for patient and medical factors known to be associated with worse ND outcomes. This study provides an understanding of neurodevelopmental sequelae beyond the infancy period and advocates for long-term ND monitoring to better understand the impact of randomization to TGC and resultant hypoglycemia over time.

## Data Availability

The datasets used and/or analyzed during the current study are available from the corresponding/senior author on reasonable request.

## Abbreviations

TGC: Tight glycemic control
ICU: Intensive care unit
ND: Neurodevelopmental
SPECS: Safe pediatric euglycemia after cardiac surgery
STD: Standard care
Bayley-III: Bayley scales of infant and toddler development, third edition
ABAS-II: Adaptive behavior assessment system, second edition
BASC-2: Behavior assessment system for children, second edition
ASQ-3: Ages and stages questionnaire, third edition
RACHS-1: Risk adjustment in congenital heart surgery (RACHS-1)
